# Haplotype-based association analysis of the MAPT locus in Late Onset Alzheimer's disease

**DOI:** 10.1186/1471-2156-8-3

**Published:** 2007-01-31

**Authors:** Odity Mukherjee, John SK Kauwe, Kevin Mayo, John C Morris, Alison M Goate

**Affiliations:** 1 Department of Psychiatry, Washington University School of Medicine, St. Louis, MO, USA; 2 Department of Neurology, Washington University School of Medicine, St. Louis, MO, USA; 3 Department of Genetics, Washington University School of Medicine, St. Louis, MO, USA

## Abstract

**Background:**

Late onset Alzheimer's disease (LOAD) is a common sporadic form of the illness, affecting individuals above the age of 65 yrs. A prominent hypothesis for the aetiopathology of Alzheimer's disease is that in the presence of a β-amyloid load, individuals expressing a pathogenic form of tau protein (*MAPT*) are at increased risk for developing the disease. Genetic studies in this pursuit have, however, yielded conflicting results. A recent study showed a significant haplotype association (H1c) with AD. The current study is an attempt to replicate this association in an independently ascertained cohort.

**Results:**

In this report we present the findings of a haplotype analysis at the *MAPT *locus. We failed to detect evidence of association of the H1c haplotype at the *MAPT *locus with LOAD. None of the six SNPs forming the H1c haplotype showed evidence of association with disease. In addition, nested clade analysis suggested the presence of independent mutations at multiple points in the haplotype network or homoplasy at the *MAPT *locus. Such homoplasy can confound single SNP tests for association. We do not detect evidence that the set of SNPs forming the H1c haplotype in general or rs242557 in particular are pathogenic for LOAD.

**Conclusion:**

In conclusion, we employed two contemporary haplotype analysis tools to perform haplotype association analysis at the *MAPT *locus. Our data suggest that the tagged SNPs forming the H1c haplotype do not have a causal role in the pathogenesis of LOAD.

## Background

Alzheimer's disease (AD [MIM 104300]) is a common, genetically influenced disorder with a prevalence rate of 5–10%. A majority of AD cases manifest as the sporadic late onset form (LOAD [MIM 606626]), typically with onset above the age of 65 years. Clinically, the disease is characterized by subtle memory loss at onset followed by a slowly progressive dementia. Pathological inclusions include β-amyloid plaques and neurofibrillary tangles (NFT) of hyperphosphorylated tau protein [1]. Many shared pathological processes such as production, aggregation, metabolism and removal of specific proteins are now recognized among neurodegenerative diseases [2]. In this context the microtubule associated protein, tau (*MAPT*) gene serves as a logical candidate gene for susceptibility for AD, however studies testing for association between polymorphisms within *MAPT *and AD have resulted in equivocal results [3-5]. Positive association studies of *MAPT *and neurodegenerative diseases divide the *MAPT *locus into two divergent clades, H1 and H2, with H2 being a single haplotype covering several genes on the long arm of chromosome 17. The H2 haplotype represents a sub-chromosomal inversion of over 1 megabase, resulting in reduced recombination in this region of chromosome 17 [6]. The H1 haplotype, on the other hand, shows considerable variation [7,8]. A recent report suggested a strong association between a specific variant of the H1 clade (H1c) with AD and other sporadic tauopathies [5,9]. In this report we sought to replicate this association in a large clinical cohort of LOAD from the same genetic pool.

## Results

### Single SNP association

**Figure 1:**
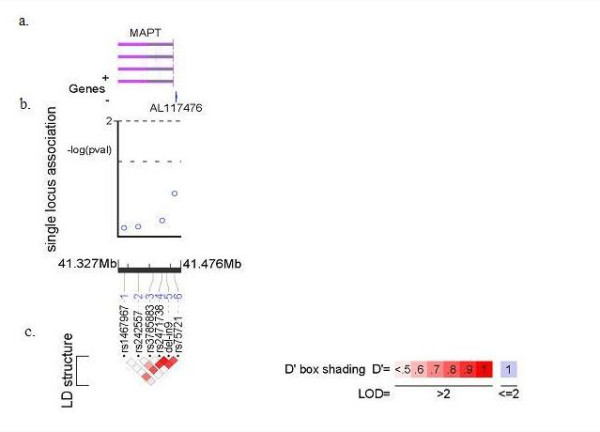
Plot of single marker association and pair-wise LD analysis of all SNPs forming the H1C haplotype. (a) The position of the markers and the ref sequence is with respect to the genome assembly hg17/May2004.(b) Association analysis of H1C haplotype SNPs with LOAD; plot of -log(P) for case-control test at the allelic level with LOAD. (c) Linkage disequilibrium plot depicting the D' value; the blocks are shaded corresponding to the values which were obtained from the LD analysis program haploview.

None of the six markers tested showed significant differences in the allelic distribution between LOAD cases and control samples. Table 1 summarizes the single SNP association results. Allele frequencies estimated using the control samples were comparable to previous published reports [5]. The del-In9 polymorphism, defining the H1/H2 clades was the only marker showing a trend towards association with AD (Table 1; fig 1b). This is in contrast to several previous reports [5,10,11].

**Table 1:** Single SNP association analysis forming the H1C haplotype at the MAPT locus.

Marker	Major allele frequency Case	Major allele frequency Control	χ^2^	P-value
rs1467967	0.66	0.64	0.2	0.63
rs242557	0.64	0.63	0.1	0.73
rs3785883	0.80	0.80	0.001	0.97
rs2471738	0.81	0.79	0.8	0.35
del-In9	0.80	0.76	3.5	0.06
rs75721	0.52	0.56	2.4	0.12

All analysis was performed using the haploview program.

### Linkage Disequilibrium analysis

Pair-wise linkage disequilibrium (LD) analysis was carried out for all of the SNPs in the 358 unrelated control individuals using the Haploview program. The position of the relevant SNPs and the resulting D' plot is presented in figure 1c. We observed high to moderate D' scores between the tagging SNPs and del-In9. Haplotype frequencies were estimated using WHAP. Most of the haplotypes inferred in this dataset matched with a previous report [5]. We did not, however, observe a difference in the haplotype frequencies between the cases and controls (Table 2).

**Table 2:** Haplotype analysis results derived using WHAP (haplotypes > 2% frequency).

Haplotype	Name	Frequency
AGGC(-)G	H2	0.230
GGGC(+)A	H1b	0.196
AAGT(+)G	H1c	0.100
AGGC(+)A	H1e	0.094
AAGC(+)A	H1d	0.073
GAGC(+)A	H1i	0.052
AGAC(+)G	H1l	0.052
AGGC(+)G		0.046
AGAC(+)A	H1h	0.040
AAGC(+)G	H1u	0.033
AAAT(+)G		0.031
GAAC(+)A		0.029
GAGT(+)G		0.025

The names of the haplotypes are as per Myers et al., (2005) [5]. Unnamed haplotypes were inferred only in this dataset. The status at del-In9 is represented by (-) for the deletion and (+) for the insertion.

### Nested Clade analysis

**Figure 2:**
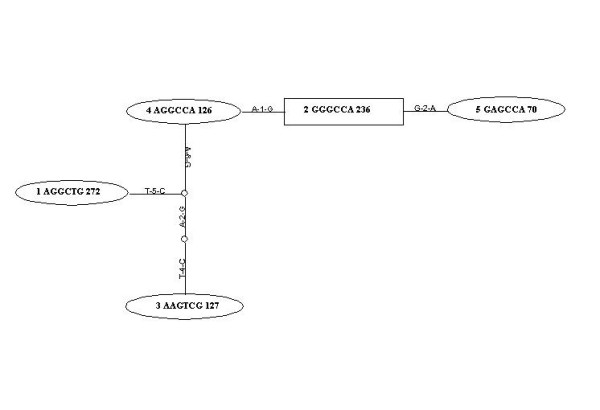
Haplotype Network of the MAPT locus: Each oval represents a specific haplotype. Branches represent mutational steps between haplotypes. The figure shows the haplotype identification number, the nucleic acid base at each locus of the haplotype and the number of times it was inferred in this sample set.

To further our analysis and confirm the above negative report; we performed an independent robust haplotype analysis which incorporates evolutionary information. HAP inferred a total of 38 phased haplotypes in our dataset. The haplotype network constructed using all of the haplotypes showed several loops indicating ambiguous connections. Due to ambiguity in the network, we did not use this network for association analysis; however we did observe homoplasy for all six SNPs. In order to decrease the ambiguity in the network it was estimated using haplotypes with a frequency of 5% or greater. The resulting network included a single ambiguous loop. Using the frequency criterion, the loop was broken and the resultant network is presented in figure 2[12]. There was no significant difference in the haplotype frequencies between the cases and the controls across any branch of the haplotype network in the nested contingency analyses (table 3). It is interesting at this point to mention that even when only common haplotypes were considered homoplasy was inferred for the promoter polymorphism rs242557.

**Table 3:** Association analysis of haplotype clades at first level of nesting.

Comparison	P value (FET*)
2 Vs 5	0.18
2 Vs 4	0.88
4 Vs 1	0.66
4 Vs 3	0.70

*FET-Fisher's exact test.

## Discussion

The assortment of conflicting results at the *MAPT *locus makes it difficult to assign a pathological role for *MAPT *in LOAD. Several reasons could contribute to this failure including methodological differences, variation in sample size and, most importantly, the use of unphased genotype data to analyze regions showing evidence of recurrent mutation and recombination. The current case control study was designed to investigate the relationship between the H1c haplotype, formed by six htSNPs in *MAPT*, with LOAD. We used a regression-based haplotype association suite as employed in the program WHAP and confirmed the negative results using a cladistic approach. Nested clade analysis provides a design to group haplotypes based on evolutionary relatedness to test for disease association [13]. This approach of grouping closely related haplotypes together increases the power of analysis by reducing the degrees of freedom. Since closely related haplotypes are grouped together, mutations of biological consequence can be localized to a small subset of haplotypes. This also guards the analysis against comparing rare haplotypes. Thus, nested clade analysis was the most appropriate method to re-evaluate the H1c association. The uncertainty of the correct haplotype network (due to ambiguity in this study) decreases the power of the approach, although it was helpful in revealing important information, such as the presence of homoplasy and historical recombination at the locus being studied. The presence of homoplasy may prove to be an important factor in the analysis of candidate regions for disease association. Although the network estimated from all the inferred haplotypes contained a large number of ambiguous connections, we were able to identify the occurrence of multiple mutations at each of these six loci at independent parts of the network. Even after removing all the rare haplotypes and constructing the haplotype network, homoplasy was observed for the marker rs242557. This marker has been previously implicated as an important risk factor for sporadic tauopathies such as PSP and CBD [9] as well as for AD [5]. The lack of association of this marker in our sample set coupled with the results of the nested clade analysis, suggests that rs242557 may not have a causal effect on AD, but may be in LD with another marker which does. It is also important to note that the samples used in this study differed from those used by Myers et al. (2005) [5]. Our samples were clinically diagnosed while the samples in their study were pathologically confirmed cases and controls. This is one possible cause for the difference in the association findings, although it would have been expected that the H1c frequency in the controls might have been higher in our study because some clinical controls may have undetected preclinical AD. However, we observed that the frequency of the H1c haplotype in our AD cases was similar to our controls and to the controls in the study of Myers et al (2005) [5].

## Conclusion

In conclusion, we used two robust haplotype analysis tools to test for association of the H1c haplotype with LOAD using a case control design. Our data does not support the positive association of htSNPs forming the H1c haplotype within MAPT with LOAD and suggests that rs242557 is unlikely to be the functional allele as previously suggested in some positive associations reported for tauopathies (Myers et al., 2005) [5].

## Methods

### Subjects

The case-control series used in this study was collected through the Washington University Alzheimer's Disease Research Center (ADRC) patient registry. Cases in this series received a diagnosis of dementia of the Alzheimer's type (DAT), using criteria equivalent to the NINCDS-ADRDA (National Institute of Neurological and Communicative Diseases and Stroke/Alzheimer's Disease and Related Disorders Association) [14], modified slightly to include AD as a diagnosis for individuals aged > 90 years [15]. A total of 361 unrelated DAT cases with a minimum age at onset of 60 years were recruited for the study. DNA from 358 age and sex matched non-demented controls aged > 60 years at assessment were obtained through the ADRC. A detailed description of the sample can be found elsewhere [16,17].

### Genotyping

A total of 6 tag SNPs, including two promoter polymorphisms (rs1467967 and rs242557), three intronic SNPs (rs3785883, rs2471738 and rs7521) and the intron 9 insertion-deletion (del-In9) polymorphism, were used in this study [5]. Written informed consent was obtained from all subjects and/or their caregiver who participated in this study. Approval from the Institutional Review Board was obtained prior to any genetic analysis. The del-In9 polymorphism was assayed by Pyrosequencing as described earlier [18]. Genotyping for the rest of the SNPs was performed using matrix assisted laser desorption/ionization time-of-flight (MALDI-TOF) mass spectrometry (Sequenom). PCR primers and primer extension assays were designed by using SPECTROGEN software (Sequenom). SNP assays were designed to generate extension products of different masses resulting in genotype dependent peak appearance.

### Statistical analysis

To measure linkage disequlibrium (LD) between the tag SNPs, Lewontin's standardized pairwise LD coefficient (D') and the Pearson's correlation (r^2^) were calculated using haploview [19]. The distribution of frequencies at the allelic and haplotypic level between cases and controls was compared using WHAP [20]. The single marker analysis is a χ^2 ^test with empirical significance with multiple testing adjustments determined by permutation. Haplotype analysis used a regression-based association test through a likelihood ratio test (LRT), which is a χ^2 ^test with n-1 degrees of freedom to determine the associated p-value.

### Nested clade analysis

Placing haplotypes in their evolutionary context improves their biological information [21]. For this analysis, phased haplotypes were reconstructed from genotype data by employing the imperfect phylogeny method implemented in the program HAP [22]. A set of 95% plausible haplotype trees, connecting the haplotypes by mutational steps, was constructed using statistical parsimony in the program TCS [23]. The presence of loops in the resulting haplotype network indicates that there are alternative, equally parsimonious ways of connecting the haplotypes. Such ambiguity in a haplotype network may be due to either recurrent mutations (homoplasy) and/or recombination within the region.

The nested statistical design proposed by Templeton and Sing (1993) was used to derive a nested design for analyzing the haplotypes [24]. The methodology involves grouping haplotypes into "clades" based on their evolutionary relatedness. Association between the phenotype and the haplotypes is performed by a series of 2 × 2 contingency tests. For each test the number of cases and controls was compared between adjacent clades using a Fisher's exact test.

## Authors' contributions

OM carried out all statistical analysis, data interpretation and drafted the manuscript. JSKK contributed to the data analysis and revision of the manuscript. KM carried out genotyping. JCM carried out the clinical assessments for the subjects enrolled in the study. AMG conceived and coordinated the study. All authors read and approved the final manuscript.
